# Augmented renal clearance: a retrospective, cohort study of urinary creatinine clearance in critically ill patients in the United Kingdom

**DOI:** 10.1177/03000605211015573

**Published:** 2021-05-26

**Authors:** Brian W Johnston, David Perry, Martyn Habgood, Miland Joshi, Anton Krige

**Affiliations:** 1 Institute of Life Course and Medical Sciences, 4591 University of Liverpool, Liverpool, UK; 2 4595 Liverpool University Hospitals NHS Foundation Trust, Liverpool, UK; 3 East Lancashire Hospital Trust, Blackburn, UK; 4 6723 University of Central Lancashire, Preston, UK

**Keywords:** Augmented renal clearance, creatinine clearance, pharmacokinetics, pharmacodynamics, prevalence, risk factor

## Abstract

**Objective:**

Augmented renal clearance (ARC) is associated with sub-therapeutic antibiotic, anti-epileptic, and anticoagulant serum concentrations leading to adverse patient outcomes. We aimed to describe the prevalence and associated risk factors for ARC development in a large, single-centre cohort in the United Kingdom.

**Methods:**

We conducted a retrospective observational study of critically unwell patients admitted to intensive care between 2014 and 2016. Urinary creatinine clearance was used to determine the ARC prevalence during the first 7 days of admission. Repeated measures logistic regression was used to determine risk factors for ARC development.

**Results:**

The ARC prevalence was 47.0% (95% confidence interval [95%CI]: 44.3%–49.7%). Age, sex, Acute Physiology and Chronic Health Evaluation (APACHE) II score, and sepsis diagnosis were significantly associated with ARC. ARC was more prevalent in younger vs. older (odds ratio [OR] 0.95 [95%CI: 0.94–0.96]), male vs. female (OR 0.32 [95%CI: 0.26–0.40]) patients with lower vs. higher APACHE II scores (OR 0.94 [95%CI: 0.92–0.96]).

**Conclusions:**

This patient group probably remains unknown to many clinicians because measuring urinary creatinine clearance is not usually indicated in this group. Clinicians should be aware of the ARC risk in this group and consider measurement of urinary creatinine clearance.

## Background

During critical illness a number of pathophysiological factors alter the pharmacokinetic and pharmacodynamic (PK/PD) handling of drugs.^1^ Alterations in hepatic enzyme activity, plasma protein concentration, and the volume of distribution (Vd) of drugs are common.^1^ Similarly, organ failure and in particular renal failure can result in reduced elimination of drugs, potentially leading to accumulation and toxicity.^2,3^

Routine monitoring of renal function has traditionally been aimed at detecting renal impairment and drugs relying on renal elimination should undergo dose adjustment to prevent toxicity.^2–4^ However, the phenomenon of a state of supra-physiological renal function known as augmented renal clearance (ARC) is increasingly recognised.^2,4^ ARC is defined as enhanced renal clearance and elimination of circulating solutes and is thought to be driven by a physiological increase in glomerular filtration rate (GFR).^5^

The presence of ARC is based upon measured urinary creatinine clearance (CrCl). CrCl values ranging from >120 mL/minute/1.73 m^2^ to >150 mL/minute/1.73 m^2^ have been used to define ARC in the literature.^2^ Despite variations in the exact CrCl value to be used, there is evidence to suggest that CrCl >130 mL/minute/1.73 m^2^ can lead to subtherapeutic plasma concentrations of drugs such as antibiotics, in particular beta-lactams, glycopeptides, and aminoglycosides, as well as anticoagulants and antiepileptic medications.^6,7^ This observation has led to a general consensus that CrCl >130 mL/minute/1.73 m^2^ is an acceptable and clinically important cut off value for the definition of ARC.^2,5,8^

A recent systematic review estimated the prevalence of ARC at 20% to 65% of admissions to the intensive care unit (ICU).^2^ Despite this high prevalence, detection of ARC in the critically unwell is challenging because it often occurs with a normal serum creatinine concentration and with little indication in biochemical markers of its presence.^9^ Furthermore, surrogate markers for renal function and estimation of GFR, such as the Cockcroft–Gault (CG), Modified CG, 4-variable Modification of Diet in Renal Disease (MDRD-4), 6-variable Modification of Diet in Renal Disease (MDRD-6), and Chronic Kidney Disease Epidemiology Collaboration (CKD-EPI) scores, tend to underestimate CrCl values and rely upon serum creatinine being in a steady state, which is not often the case in critically unwell patients.^10,11^ Poor correlation with estimated GFR coupled with few ICUs routinely measuring CrCl has led to the introduction of a number of scoring systems aimed at improving the detection of ARC.^2,12^ Such scoring systems may help to identify patients in which formal CrCl measurement may be beneficial to assess for ARC and guide drug therapy and dose adjustment for renally cleared medications.

In 2014 our institution introduced continuous antibiotic infusions with daily dose adjustment guided by routine daily measurement of CrCl, thus providing a rich data source of renal function across a broad cohort of critical care admissions. To the best of our knowledge there are few data in the literature regarding the prevalence of ARC and its associated risk factors in a British critical care population. In the present study, we aimed to examine the prevalence of ARC, determine risk factors associated with ARC, investigate the development of ARC during the first 7 days of critical illness, and assess whether the Augmented Renal Clearance in Trauma Intensive Care (ARCTIC) score can be used as a screening tool for ARC in a cohort of mixed surgical and medical patients admitted to an ICU in the United Kingdom.

## Methods

### Study setting

We conducted a retrospective observational cohort study of patients admitted to the ICU of the Royal Blackburn Teaching Hospital between January 2014 and December 2016. The Royal Blackburn Teaching Hospital is a 24-bed tertiary referral centre for patients with hepatobiliary illnesses which has an increased proportion of young patients with pancreatitis compared with similar sized institutions. It is not a regional trauma centre and therefore admits comparatively few trauma patients. The study was granted Health Research Authority approval on 2 February 2017 (IRAS project ID 220861) following proportionate ethical review as the study analysed clinical data routinely collected by the clinical team which were then anonymised. Therefore, the requirement for individual patient consent was waived for this study.

### Patient selection

Patients were eligible for inclusion if they were an inpatient in the ICU, were over 18 years of age, and had a CrCl value measured within 24 hours of admission. Patients were excluded from the analysis if they suffered acute kidney injury (AKI), defined as serum creatinine > 110 mmol/L on or during admission; had documented Kidney Disease: Improving Global Outcomes (KDIGO) stage 5 chronic kidney disease (CKD) or end-stage renal failure (ESRF); or underwent renal replacement therapy (RRT) during ICU admission.^13^

### Data collection and definition of ARC

Data were extracted from electronic medical records and anonymised prior to analysis. Demographic data including age, sex, height, weight, diagnosis of diabetes mellitus, and presence of KDIGO stage 5 CKD or ESRF were recorded. Acute Physiology and Chronic Health Evaluation (APACHE) II scores, length of ICU stay, level of cardiovascular support, and admission diagnosis were extracted from the ICU Wardwatcher database. This database captures data on all critical care admissions for transmission to the Intensive Care National Audit and Research Centre (ICNARC). Admission diagnosis was coded as 1) sepsis, 2) post-operative patients without sepsis, 3) pancreatitis 4) trauma, or 5) other. Daily serum creatinine levels, daily blood glucose levels, and daily 6-hourly CrCl levels were extracted from electronic laboratory results, and 6-hourly urine samples were collected from an indwelling urinary catheter daily between 2200 and 0400. Urinary creatinine measurement was undertaken on the entire 6-hour diuresis and utilised automated analysers employing the enzymatic Ortho Vitros method (Ortho Clinical Diagnostics, Raritan, NJ, USA). Creatinine clearance was calculated using the standard formula: CrCl (mL/minute) = (6 hourly urinary volume (mL) × Urinary_Cr_ (mmol/L)/(Plasma_Cr_ (µmol/L) × 360 minutes) and was corrected for body surface area (BSA) using the formula: Corrected CrCl = CrCl × (1.73 m^2^/BSA)^2^. BSA was estimated using the Du Bois formula: BSA = 0.007184 × weight (kg)^0.425^ × height (cm)^0.725^ ^14^. The presence of ARC was defined as CrCl > 130 mL/minute/1.73 m^2^.

### Outcomes

Our primary outcome of interest was to define the overall prevalence and prevalence for the development of ARC on each day of the first 7 days of ICU admission. Secondary outcomes were to identify independent risk factors associated with the development of ARC and to assess the predictive ability of the ARCTIC score to predict ARC in our patient cohort.

### Statistical analysis

All data were de-identified and anonymised prior to analysis. Baseline and measured study characteristics are presented as medians and interquartile ranges (IQR) for continuous variables and as counts (%) for categorical variables.

The prevalence of ARC with 95% confidence intervals was calculated as the number of patients with ARC out of the number of patients with a recorded CrCl value. Overall prevalence was defined as any patients developing ARC during admission, and the daily prevalence over the first 7 days of admission was calculated. Occurrence of ARC was modelled as a binary response. Repeated measures on individuals were available, and our area of interest was the population rather than individual prediction. Accordingly, we used repeated measures logistic regression with possible risk factors as explanatory variables, and fitted a population-averaged model. Candidate explanatory variables used in univariate analysis included age, height, weight, BSA, ICU length of stay, level of cardiovascular support, APACHE II score, sex, admission serum glucose levels, APACHE II mortality, and admission diagnosis. Explanatory variables for the model were selected using backward selection with a cut-off p-value of 0.05 signifying significance.

Odds ratios were calculated and are reported for significant explanatory variables. We assessed the ARCTIC scoring system in predicting ARC.^14^ We limited the analysis to day 1 of admission because the ARC status of our cohort was not constant throughout admission. All statistical analyses were carried out using STATA 16 software (StataCorp LP; College Station, TX, US).

Reporting of our study conforms with broad EQUATOR guidelines, and specifically with the STROBE checklist for retrospective studies.^15^

## Results

**Figure 1.**
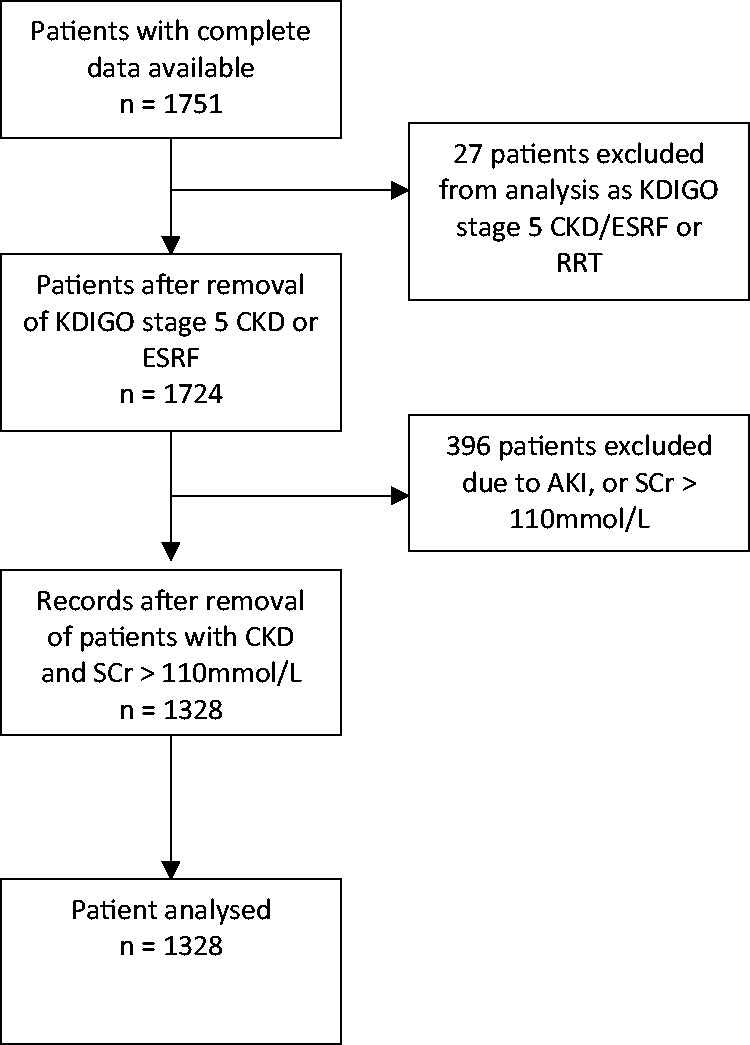
Flow diagram of patients included in logistic regression analysis. KDIGO, Kidney Disease: Improving Global Outcomes; CKD, chronic kidney disease; ESRF, end-stage renal failure; RRT, renal replacement therapy; AKI, acute kidney injury; SCr, serum creatinine.

During the 2-year study period, complete data were available for 1751 individual adult patients. Following exclusion of patients who were admitted with or developed AKI during their ICU stay, required RRT, or had documented KDIGO stage 5 CKD or ESRF, data for 1328 individual patients were available for analysis (Figure 1).

### Patient characteristics

The median age of our patient cohort was 64 years (IQR 50–73) with more male (714 (53.7%)) than female (614 (46.2%)) patients. Our study cohort had a median weight of 74 kg (62–87.5 kg) and a median height of 1.68 m (1.60–1.75 m). The median length of ICU stay was 3.3 days (1.9–6.8 days).

The majority of the patients included (1037, 78.1%) had no previous diagnosis of diabetes mellitus. The main characteristics of our entire patient cohort are presented in Table 1.

**Table 1.** Demographic, admission, and illness severity data in patients with and without ARC (at any time).

	Whole cohort (n = 1328)	ARC (n = 624)	Non-ARC (n = 704)	p-value
Age (years), median (IQR)	64 (50–73)	56 (43–68)	68 (58–78)	<0.001
Sex, male, n (%)	714 (53.8%)	406 (65.0%)	308 (43.8%)	<0.001
Sex, female, n (%)	614 (46.2%)	218 (35.0%)	396 (56.2%)	<0.001
Height, median (IQR)	168 cm (160–175 cm)	171 cm (164–178 cm)	165 cm (157–173 cm)	<0.001
Weight, median (IQR)	74 kg (62–87.5 kg)	81 kg (68–93 kg)	68 kg (59–80 kg)	<0.001
BSA (m^2^), median (IQR)	89.2 m^2^ (73–108 m^2^)	99.6 (81.8–116.2)	81.7 (68.6–97.1)	<0.001
Diabetes mellitus n (%)	291 (21.9%)	73 (25%)	218 (74%)	0.006
APACHE II score, median (IQR)	14 (11–18)	13 (10–16)	15 (12–19)	<0.001
Length of stay, n, (IQR)	3.30 days (1.88–6.79 days)	3.80 (1.91–7.67)	3.06 (1.85–6.05)	<0.001
Admission diagnosis, n (%)				
Sepsis	349 (26.2%)	145 (23.2%)	204 (29.0%)	0.017
Post-operative without sepsis	419 (31.5%)	183 (29.3%)	236 (33.5%)	0.10
Pancreatitis	29 (2.1%)	22 (3.5%)	7 (1.0%)	0.002
Trauma	29 (2.1%)	15 (2.4%)	14 (2.0%)	0.62
Other	502 (37.8%)	259 (41.5%)	243 (34.5%)	0.009
KDIGO renal classification between patients with and without ARC				
eGFR >90 mL/minute/1.73 m^2^		623 (99.8%)	678 (96.3%)	1
eGFR 60–90 mL/minute/1.73 m^2^		0 (0.0%)	1 (0.14%)	0.35
eGFR 45–59 mL/minute/1.73 m^2^		0 (0.0%)	2 (0.28%)	0.19
eGFR 30–44 mL/minute/1.73 m^2^		1 (0.16%)	20 (2.84%)	<0.001
eGFR 15–29 mL/minute/1.73 m^2^		0 (0.0%)	3 (0.43%)	0.10

ARC, augmented renal clearance; IQR, interquartile range; BSA, body surface area; APACHE II, Acute Physiology and Chronic Health Evaluation II; KDIGO, Kidney Disease: Improving Global Outcomes; eGFR, estimated glomerular filtration rate.

### Prevalence of ARC

The adjusted prevalence of ARC in our cohort was 47.0% (95% CI = 44.3%–49.7%). Within this cohort, 624 or 47% (95% CI = 44.3%–49.7%) had ARC at some point during their ICU stay, 272 or 20.5% (95% CI = 18.4%–22.7%) had ARC throughout their stay, which never resolved; a further 185 or 13.9% (95% CI = 12.2%–15.9%) had ARC that resolved at some point during the ICU stay, and 167 or 12.6% (95% CI 10.9%–14.5%) had intermittent ARC that was unresolved during the ICU stay. For patients remaining in the ICU for 7 days or longer, the prevalence of ARC ranged from 22.1% to 24.9% over the first 7 days (Table 2). The median time to onset of ARC was 1 day with over 64% of patients that developed ARC doing so within 24 hours.

**Table 2.** Daily prevalence of ARC over the first 7 and subsequent days (n = 1328).

Day	ARC n (%)	Non-ARC n (%)
1	281 (45.8%)	333 (54.2%)
2	98 (45.6%)	117 (54.4%)
3	52 (40.0%)	78 (60.0%)
4	46 (63.0%)	27 (37.0%)
5	20 (40.8%)	29 (59.2%)
6	17 (50.0%)	17 (50.0%)
7	14 (50.0%)	14 (50.0%)
8+	96 (51.9%)	89 (48.1%)

ARC, augmented renal clearance.

**Figure 2.**
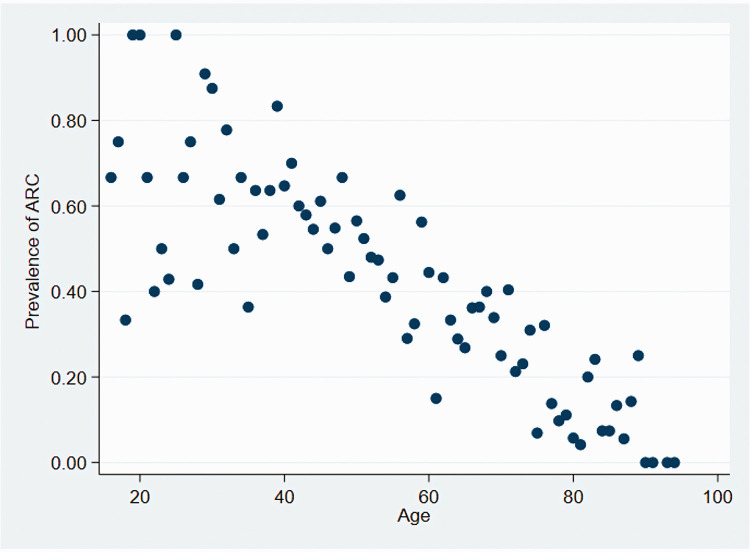
Scatter plot showing the prevalence of ARC versus age in patients that developed ARC at any point during admission. ARC, augmented renal clearance.

ARC was more common among men with a prevalence of 36.9% compared with 35.5% among women (p<0.001). Patients that developed ARC tended to be significantly (p<0.001) younger (median age 56 years) compared with those that did not develop ARC (median age 68 years). We could not identify an age threshold below which ARC was more likely; however, the likelihood of developing ARC decreased with increasing age (Figure 2).

Patients with ARC had a median length of ICU stay of 3.8 days (IQR 1.9–7.7 days) compared with 3.1 days (IQR 1.9–6.1 days) for those without ARC. Patients with ARC had a larger median BSA of 100 m^2^ (IQR 82–116 m^2^) compared with 82 m^2^ (IQR 69–97 m^2^) for patients without ARC. Median APACHE II score was 14 (11–18). Patients without ARC were more unwell with median APACHE II score of 15 (12–19) compared with 13 (10–16) for those with ARC. Comparisons between patients with and without ARC are shown in Table 1.

### Risk factors and prediction of ARC

With logistic regression prior to backward selection of the promising explanatory variables, age (OR 0.95 [95% CI = 0.94–0.96, p<0.001]) and sex (OR 0.52 [95% CI = 0.40–0.69, p<0.001]), high glucose (defined as glucose >12–41 mmol/L) in the first 24 hours (OR 0.98 [95% CI = 0.95–1.00, p<0.027]), and date of onset of ARC within 24 hours where significantly associated with development of ARC (Table 3). There was weak evidence for APACHE II score (OR 0.97 [95% CI = 0.94–1.01, p = 0.11]) and correlation with development of ARC. No other explanatory variables (height, weight, BSA, length of stay, level of cardiovascular support, low glucose levels, admission diagnosis) were significantly associated with the development of ARC in unadjusted analysis (Table 3).

**Table 3.** Population-averaged risk factors for ARC in model before backward selection.*

Variable	Odds ratio (95% CI)	p-value
Unadjusted population-averaged risk factors for ARC		
Age	0.949 (0.942–0.956)	<0.001
Sex	0.523 (0.397–0.689)	<0.001
Height	1.030 (0.986–1.075)	0.188
Weight	1.030 (0.943–1.125)	0.515
BSA	0.997 (0.927–1.072)	0.937
Length of stay	1.018 (0.977–1.060)	0.393
APACHE II score	0.972 (0.939–1.006)	0.105
APACHE II mortality prediction	0.995 (0.983–1.006)	0.339
Advanced Cardiovascular support	0.957 (0.871–1.051)	0.359
Low glucose within first 24 hours	1.039 (0.983–1.099)	0.179
High glucose within first 24 hours	0.972 (0.948–0.997)	0.027
Date of onset of ARC	0.999 (0.999–0.999)	0.018
Adjusted population-averaged risk factors for ARC after backward selection		
Age	0.951 (0.944–0.958)	<0.001
Sex	0.518 (0.393–0.685)	<0.001
Height (cm)	1.043 (1.030–1.057)	<0.001
BSA	0.981 (0.964–0.998)	0.001
APACHE II score	0.953 (0.932–0.974)	<0.001
Low glucose within first 24 hours	1.057 (1.000–1.169)	0.048
High glucose within first 24 hours	0.977 (0.953–1.001)	0.062
Date of onset of ARC	0.999 (0.999–0.999)	0.011
Diagnosis of sepsis	0.764 (0.592–0.984)	0.03

*Except for renal diagnosis code, with which the model would not converge as there were no values apart from zero with arc=1, and the term was non-significant in the model.

ARC, augmented renal clearance; CI, confidence interval; BSA, body surface area; APACHE II, Acute Physiology and Chronic Health Evaluation II.

Following backward logistic regression for multiple explanatory variables, the significant associations were preserved. In particular, ARC was found to be significantly associated with younger vs. older age (OR 0.95 (95% CI = 0.94–0.96, p<0.001)), male vs. female sex (OR 0.52 (95% CI = 0.39–0.69, p<0.001)), and diagnosis of sepsis (OR 0.763 (95% CI = 0.592–0.984 p=0.037)). APACHE II score, while not significant in unadjusted analysis, was significant following adjusted analysis, with a lower vs. higher APACHE II score being associated with ARC (OR 0.95 [95% CI = 0.93–0.97, p<0.001]).

In assessing the ARCTIC scoring system we used ARC status of CrCl >130 mL/minute/1.73 m^2^ and found that the ARCTIC score had a sensitivity of 0.72 (95% CI = 0.67–0.76) and specificity of 0.63 (95% CI = 0.60–0.66) for predicting ARC. We found that of 565 patients having a positive ARCTIC score (6 or more), 289 had ARC, giving a positive predictive value of 51.1% (95% CI = 47%–55%). Of the 585 patients with negative ARCTIC score, 473 did not have ARC, giving a negative predictive value of 80.9% (95% CI = 77.5%–83.8%).

## Discussion

In this retrospective observational study, we have shown that ARC is a frequent occurrence in a large cohort of 1328 patients in a mixed medical and surgical ICU. The overall prevalence of 47.0% highlights that a significant number of patients develop ARC during admission to ICU. Our observed prevalence is in keeping with that of similar observational studies estimating that 20% to 65% of critically ill patients will develop ARC at some point during ICU admission.^5,4,16–19^ In patients with ARC in our analysis, 20.5% remained in ARC status throughout their admission. Similar findings have been reported by De Waele *et al.*,^20^ who showed that ARC was permanently present in 23% of patients but transient in 35% of mixed medical and surgical patients admitted to ICU. Grootaert *et al.* found that 40% of patients that developed ARC had CrCl > 120 mL/minute/1.73 m^2^ for at least 5 days while Udy *et al.* and Fuster–Lluch *et al.*^4,21,22^ reported that the highest prevalence of ARC was on day 5 after admission to ICU.

We found that age, sex, APACHE II score, and diagnosis of sepsis were significantly associated with ARC. ARC was present in younger (56 vs. 68 years), male patients with lower APACHE II scores (13 vs. 15). Younger age has been consistently linked with ARC in a number of studies, and a recent systematic review reported an average difference of 10 years between patients with and without ARC.^2,8,9,23^ ARC is rarely found in patients over 50 years, and Ruiz *et al.*^2,24^ reported using <58 years as a screening cut off for ARC. While our findings did not establish an age threshold above which ARC was absent, there was a clear trend towards fewer patients with ARC as age increased. GFR decreases with age, with studies estimating a decline of 8 mL/minute/1.73 m^2^ during each decade after the age of 40 years.^25^ A number of reasons for this decline have been postulated, including reduction in glomerular capillary plasma flow rate through reduction in afferent arteriolar resistance and increased glomerular hydraulic pressure resulting in reduced GFR.^25^ Coupled with structural changes associated with aging such as reduced renal mass and tubulointerstitial fibrosis, it is likely that a reduction in GFR accounts for the trend in reduced ARC with increasing age observed in our cohort.^25^

The association between ARC and younger age likely explains the relationship between ARC and illness severity scores such as APACHE II and the Simplified Acute Physiology Score (SAPS-II) as well as the shorter ICU length of stay in the patients with ARC in our cohort. In multiple studies, lower APACHE II or SAPS-II score has been shown to be a risk factor for the development of ARC.^2,5,6,16,26^ However, this relationship has not been observed in studies that used the Sequential Organ Failure Assessment (SOFA) score, which does not include age as a variable.^23^ Kawano *et al*., in a study of 111 patients, revealed that APACHE II score but not SOFA score was associated with ARC when both scores were measured in the same cohort of patients, and reported that this difference following multivariate analysis could likely be explained by the influence of age on APACHE II and SAPS-II.^23^ In our study, it is plausible that the inclusion of age in the APACHE II score is responsible for the observed association with ARC.

In common with other studies, we found that ARC was more prevalent in male patients.^17,26^ Baptista *et al.*^18^ reported that ARC was more common in male trauma patients with lower SOFA scores and requiring vasopressor support. While our cohort displayed more cases of ARC among male patients, we did not find a significant association between ARC and vasopressor use or the requirement for advanced cardiovascular support. Trauma has been described as a risk factor for ARC in multiple studies, although the mechanism by which trauma leads to ARC remains unclear.^6,23,24,27^ Our ICU admits relatively few trauma patients, yet the association between ARC and younger male patients remained, suggesting that the association between trauma and ARC may actually reflect the demographic of patients that are more likely to present following trauma rather than a true physiological influence.^4,28^ Despite this, trauma has been shown to be a significant risk factor in a number of multivariate analyses, and Barletta *et al.*^16,17,24^ recently developed a scoring system for use in trauma patients to predict ARC. The ARCTIC scoring system has previously been reported to predict ARC with a sensitivity of 0.84 and specificity of 0.68 in 133 trauma patients by combining variables that we also found to be significantly associated with ARC, such as age, male sex, and low serum creatinine levels.^17^ In our cohort we found that the ARCTIC score had a sensitivity of 0.72 (95% CI = 0.67–0.76) and specificity of 0.63 (95% CI = 0.59–0.66), with a positive predictive value of 51.1% (95% CI = 47%–55%) and negative predictive value of 80.9% (95% CI = 77%–83%). Taken together, these data suggest that the ARCTIC score may be a valuable test for exclusion rather than inclusion. However, it should be noted that the ARCTIC score was developed for use in trauma patients, and that only 2% of our cohort were trauma patients. Despite the low accuracy of the ARCTIC score, the development and validation of more accurate clinical scoring systems may provide a means of identifying patients at higher risk of developing ARC, thus allowing targeted measurement of CrCl; such methods warrant further investigation to avoid reliance upon estimated GFR.

The development of ARC significantly impacts the PK of many drugs and potentially leads to alterations in plasma concentrations of renally excreted drugs important in ICU such as antibiotics, enoxaparin, and anti-epileptics.^2,7^ This situation may not be immediately obvious as few ICUs routinely measure CrCl and estimated measures of GFR have been shown to correlate poorly with CrCl.^2,12,18^ Our ICU admits predominantly post-operative patients, patients with sepsis, and patients with pancreatitis. A significant proportion of these patients will receive antibiotics either peri-operatively or as part of treatment. In those patients developing ARC, there is a risk that significant numbers of patients may not attain therapeutic plasma antibiotic concentrations without dose adjustment to match their renal function. Subtherapeutic delivery of antibiotics is likely in turn to negatively impact critical care outcomes.

Several recent guidelines, including the Surviving Sepsis Guidelines, have recommended dosing antibiotics based upon PK/PD properties of antimicrobial drugs.^29^ These guidelines highlight that therapeutic underdosing is common in critically ill patients and that ARC may play a role in this observation.^29,30^ The clinical heterogeneity of sepsis and variability of the population make it difficult to achieve individualised antibiotic plasma concentrations and may help explain why it has been difficult to establish a relationship between ARC and clinical outcome.

A significant number of patients in our cohort were admitted postoperatively. Although it was not possible to determine how many patients were admitted electively, we anticipate that a proportion of these patients were admitted following elective surgery. Elective post-operative patients are usually less unwell, have fewer organ failures, and have better physiological and haemodynamic parameters compared with patients admitted non-electively to ICU. While it is unlikely that these patients were admitted for longer than 24 hours post-operatively, it is possible that inclusion of these patients may have skewed our data towards patients with a shorter length of ICU stay, lower APACHE II score, younger age, and reduced renal dysfunction. Despite this limitation, significantly more of our patients were admitted non-electively. Unfortunately, our hospital diagnosis coding does not allow us to determine the reasons for admission beyond what is presented. We acknowledge that a significant proportion of patients included in our study had a diagnosis of, ‘other.’ It is therefore a limitation of our study that we cannot further categorise these admission diagnoses to elucidate the impact of diagnosis on ARC. Future studies should ensure a high granularity of data collection and diagnoses to ensure that the impact of individual diagnosis on ARC is investigated. An important population to consider is those elective patients admitted post-operatively, who represent an important subgroup in which the effect of ARC on the PK/PD of drugs may be particularly relevant as these patients tend to be younger and healthier and will often have prophylactic antibiotics administered intraoperatively and perioperatively. Our study is limited by not reporting antibiotic concentrations with which to investigate the impact of ARC; however, to our knowledge, few ICUs titrate antibiotics according to plasma concentrations and this is an area of study consistently lacking in the literature. Specifically designed studies aimed at defining the impact of ARC on patient outcomes and measures to individualise patient therapy based upon PK/PD measures are urgently needed.

## Conclusion

To the best of our knowledge, our study represents the largest cohort of patients with ARC admitted to intensive care in the United Kingdom and one of the largest cohorts of patients with ARC in the literature. We report an ARC prevalence of 47% in our cohort. The prevalence of ARC was greater among younger (vs. older) male patients with lower (vs. higher) APACHE II scores. Clinicians in ICUs need to be alert to the possibility of ARC and the requirement for urinary CrCl measurement for its diagnosis. This is particularly important in patients at greater risk of ARC. Further studies are required to fully understand the effects of ARC on clinical outcomes.
